# Editorial: Regulatory Action of Calcium Channels in Pain Pathway

**DOI:** 10.3389/fncel.2021.701105

**Published:** 2021-05-31

**Authors:** Senthilkumar Rajagopal, Sengottuvelan Murugan, Divya P. Kumar, Girish S. Kesturu, Albert Baskar Arul

**Affiliations:** ^1^Department of Biochemistry, Rayalaseema University, Kurnool, India; ^2^Department of Animal Sciences, School of Environmental and Biological Sciences, Rutgers, The State University of New Jersey, New Brunswick, NJ, United States; ^3^Department of Biochemistry, Jagadguru Sri Shivarathreeshwara University (JSS) Medical College, Center of Excellence in Molecular Biology and Regenerative Medicine (CEMR), Jagadguru Sri Shivarathreeshwara University (JSS) Academy of Higher Education and Research, Mysore, India; ^4^Department of Biochemistry, Tumkur University, Tumkur, India; ^5^RASR Laboratory, Department of Chemistry, Vanderbilt University, Nashville, TN, United States

**Keywords:** calcium channels, pain, G protein couple receptors, diabetic neurapathies, inflammatory

Pain is a distressing feeling often caused by intense or damaging stimuli. Pain is classified into three categories as nociceptive, neuropathic, and inflammatory (Bourinet et al., 2014). Acute pain can be mild and lasts for few seconds to minutes. Chronic pain is a pain that is ongoing and usually lasts longer than 6 months. A wide range of different types of voltage- and ligand-gated ion channels, including sodium, calcium, and TRP channels and other channels are critically involved in the detection and processing of painful stimuli in sensory neurons. The schematic diagram (Figure 1) depicts the involvement of various calcium channels in mediating pain signaling. Pain mediators such as bradykinin, serotonin, substance P, and prostaglandin E2 increase Ca^2+^-influx through Cave leading to a significant increase in intracellular calcium [(Ca^2+^)i]. The elevated [(Ca^2+^)i] signal can contribute to increased neural activity that is relayed to the central nervous system leading to increased pain perception (Senthilkumar et al., 2010; Patel et al., 2017). Previous studies have shown that chronic changes in the expression of ion channels and function contribute to chronic pain to some extent. These ion channels mediate cell signaling as well as regulate membrane potential and excitability functions, which include the release of neurotransmitters, the activation of calcium-dependent enzymes, and calcium-dependent changes in plasticity and gene transcription (Senthilkumar et al., 2010; Senthilkumar and Murugavel, 2017).

**Figure 1:**
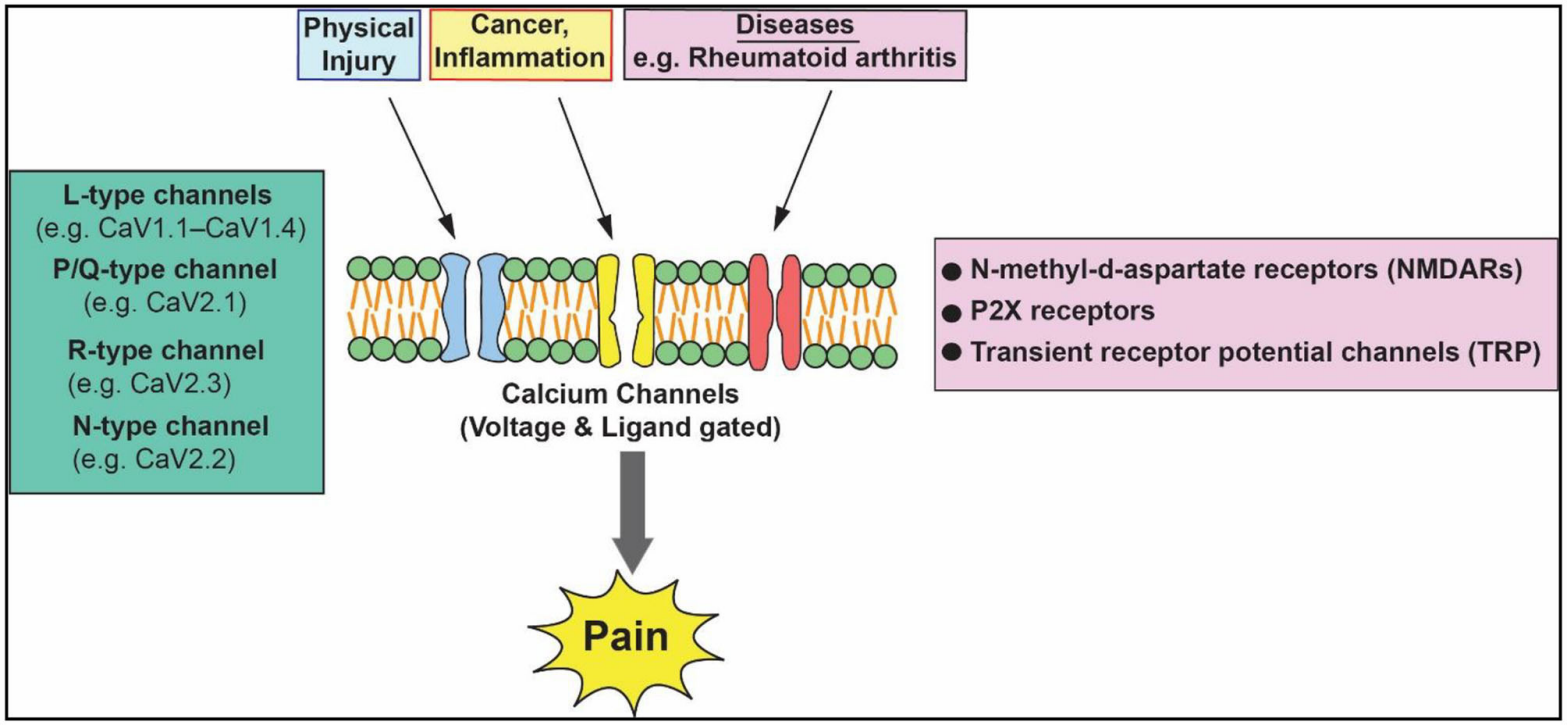
Regulatory action of volltage aged calcium channels in pain pathway.

This special issue entitled “Regulatory Action of Calcium Channels in Pain Pathway” features review articles, perspective, and an original article that sheds light on the role of calcium channels in the modulation of pain signals in health and disease.

Joksimovic et al. have provided experimental evidence of how post-translational modification such as glycosylation adversely affects the biophysical property of Ca_V_3.2 channels leading to the development of hyperalgesia in type 1 peripheral diabetic nephropathy. Furthermore, it has been demonstrated that deglycosylation caused by the administration of glycosylation inhibitors (neuraminidase and PNGase-F) potentially ameliorated the pain in STZ (streptozotocin)–treated diabetic animals (both mice and rats). Cho and Huh have reviewed the literature extensively and discussed how calcium channels in astrocytes play a critical role in chronic pain development. The role of various calcium sources and their contribution to different types of reactive astrocytes, which result in having a differential effect on chronic pain are well-described signifying how astrocytic calcium may offer better targets for pain control.

Muller and Reggio in the perspective article have analyzed the putative cannabidiol (CBD) binding site in the transient receptor potential (TRP) ion channels. CBD, a cannabinoid ligand is known to modulate different types of TRP channels such as TRPV1, TRPV2, TRPV3, TRPV4, TRPA1, and TRPM8 channels that are implicated in inflammation and chronic pain. The study clearly describes the differences that exist in the putative CBD binding sites and how this can be exploited in targeting the endocannabinoid system for the modulation of pain. Alles et al. have discussed the role of pregabalin, a gabapentinoid that exhibits a high affinity to voltage-gated calcium channels. The review article discusses the alpha 2 delta (α2δ) subunits of calcium channels in pathophysiology and how pregabalin selectively binding to α2δ1 and α2δ2 subunits performs its action to alleviate acute and chronic pain. Thus, highlights the potential therapeutic role of pregabalin in the treatment of chronic pain.

Taken together, this special issue aims to provide an overview of the different calcium-permeable ion channels involved in pain processing pathways.

## Author Contributions

All authors listed have made a substantial, direct and intellectual contribution to the work, and approved it for publication.

## Conflict of Interest

The authors declare that the research was conducted in the absence of any commercial or financial relationships that could be construed as a potential conflict of interest.
